# Behavioral pattern of Rohilkhandi kids under different feeding systems

**DOI:** 10.14202/vetworld.2016.773-776

**Published:** 2016-07-27

**Authors:** Anjali Kumari, B. H. M. Patel, Vipin Maurya, Asu Singh Godara, Med Ram Verma, Mukesh Singh

**Affiliations:** 1 Livestock Production and Management Section, Indian Veterinary Research Institute, Izatnagar, Bareilly, Uttar Pradesh, India; 2 Division of Livestock Economics Statistics and Information Technology, Indian Veterinary Research Institute, Izatnagar, Bareilly, Uttar Pradesh, India

**Keywords:** agonistic behavior, chopped, feeder, goat, growth

## Abstract

**Aim:**

The present study designed to evaluate the effect of different feeding systems on the behavior of local Rohilkhandi kids.

**Materials and Methods:**

A total of 21 growing goats (local goat of Rohilkhand region), weighing around 7-11 kg and aging 4-5 months, were used. These animals were kept in three groups. Group I was fed un-chopped green fodder in circular feeder (newly designed). Group II was fed un-chopped green fodder in linear feeder that was similar to the existing farm practice. Group III was fed chopped green fodder in linear feeder (modified version). Amount of concentrate and dry fodder fed was kept constant for all the three groups subject to equal increment in accordance with their increasing age. *Adlibitum* green fodder was made available to the animals. The experiment was conducted for 3 months. On-going behavior was recorded each day 4 h (2 h in the morning from 9:00 am to 11:00 am, after offering the feed, and same was repeated for 2 h in the afternoon, i.e., from 2:00 pm to 4:00 pm) was made between 9 am and 5 pm. The individual behaviors, *viz*., feeding, drinking, lying down, ruminating, idling, butting, pressing, pushing, frontal clashing, and physical displacement at feed barrier (active and passive: Without physical contact) of the goat were recorded using time-sampling method. Further, incidental activities such as defecation and urination were also recorded.

**Results:**

Among all the groups, butting, head to head, and pushing were the common agonistic behavior found but values did not differ significantly. The pushing while feeding was relatively less in Group II (0.22±0.04 min) which differed significantly (p<0.05) from the other two groups. The idling time was found significantly (p<0.05) lower in Group II (1.68±0.21) as compared to Group I (4.67±0.52) and Group III (4.27±0.56). Time spent in rumination near the feeding trough as well as away from the feeding trough was also significantly higher in Group I (p<0.05) than the other two groups. Other minor activities, viz., defecation and urination were negligible. No stereotypic activities were observed.

**Conclusion:**

It was concluded that provision of un-chopped fodder in circular feeder could only simulate natural feeding behavior of goat but did not give any added advantage. Further, feeding chopped fodder in linear feeding trough lead to increased consumption and more time is spent on feeding than on agonistic behavior as compared to the other two groups.

## Introduction

Goat is being reared under different rearing systems depending on the region, breed, and type of farmer. Under urban and peri-urban areas, intensive system/confinement is the only option due to the scarcity of space under which the goats are exclusively stall fed (zero-grazed). Whether intensive or semi-intensive system, there should be some arrangement for feeding of goats as per the browsing behavior of the animals. Goats naturally prefer to eat at the height of about 20-120 cm above the ground [1] in standing position. Unlike sheep and cattle, which predominantly select leafy material during spring, browse constitutes 50-80% of the forage selected by goats all year round [2]. Goats can overcome physical defenses of certain trees because they have hard mouth parts that are unaffected by spines and thorns [3].

Most of the feeders available in the market are mostly linear or hexagonal. Similarly, earlier researchers [4] have attempted to feed the green fodder in chopped form, but they found that it resulted in variable intakes. In addition to variable intake and growth, behavioral response to new feeding system is also equally important. In goats, agonistic behavior can be expressed as aggression with contact, i.e., biting, bumping, or aggression without contact, i.e., threat displays, chases, and escapes [5]. During feeding, aggressive postures in goats can include side-on locking of horns, butting the flank of another feeding goat, and ear biting [6]. It is not, therefore, surprising that aggressive interactions occur frequently among loose housed goats during feeding [7]. Recent observations in larger groups of horned and hornless goats suggest that distinct differences in social behavior are more reflected in feeding place occupancy in small groups due to the overall small pen dimensions [8].

Keeping above points in view, we have attempted to compare the behavior of goats by feeding the un-chopped fodder in newly designed feeding trough and chopped fodder in modified linear feeder.

## Materials and Methods

### Ethical approval

The experiment was duly approved by the Institutional Animal Ethics Committee, ICAR - Indian Veterinary Research Institute (IVRI), Izatnagar, Bareilly, Uttar Pradesh.

### Study site

The present study was conducted on local Rohilkhandi kids by different feeding systems for 3 months, i.e., November 2014 to January 2015 at the Sheep and Goat Farm of Livestock Production Management section, ICAR - Indian Veterinary Research Institute Izatnagar, Bareilly (Uttar Pradesh) in the northern part of India. Sheep and goat farm is located at an altitude of 250 m above the mean sea level at 29.42°N latitude and 79.54°E longitude in western Uttar Pradesh, which comes under the Upper-Gangetic Plain Agro-Climatic Zone of India. Meteorological conditions include subtropical weather (hot, humid in summer, and near freezing temperature in winter), average annual rainfall approximately 760-960 mm (received mostly during the months of July and August), and relative humidity 41-85%. There are four major seasons that prevail in a year, *viz*., winter (December-March), summer (April-June), rainy (July-September), and autumn (October-November).

### General management

A total of 21 growing goats of 7-11 kg body weights (average body weight 8.5 kg) and age 4-5 months were made into three groups consisting seven animals in each group.

#### Group I (circular)

All goats under this group were maintained on un-chopped fodder. This un-chopped fodder was fed using the circular feeder. This circular feeder was newly developed by farm workshop, IVRI. This measured 94 cm in diameter (lower) and 168 cm in height (total). This feeder was sufficient for feeding at least 7 goats. This feeder design was supposed to discourage fight among the goats due to less contact of body and ease of leaving the feeding place. Furthermore, this feeder design exploited the natural browsing behavior of goats.

#### Group II (linear)

The un-chopped fodder was fed in the linear feeder which measured 240 cm in length, 54 cm in breadth, and 88 cm in height. This feeder was being used since long time in the farm.

#### Group III

Goats were fed chopped fodder in linear feeder having length 153 cm, breadth 46 cm, and height 88 cm. However, the feeder was modified in such a way that only head of the animal could get into the manger not the whole animal to avoid contamination with feces and urine. The length of chopped fodder was 1-2 inch for maize during the experimental period.

All the three groups were fed the same amount of green fodder. Amount of concentrate and dry fodder (wheat straw) fed was kept constant for all the three groups subject to equal increment in accordance with their increasing age. The composition of concentrate was grain (maize + barley) - 25%; wheat bran - 47%; cake (deoiled soyabean cake + mustard oil cake) - 25%; mineral mixture - 2%; salt - 1%. The experiment was conducted for 3 months. On-going behavior was recorded for 2 h in the morning, and after offering the feed, and same was repeated for 2 h in the afternoon. In each group, 4 animals were observed consecutively for 4 days. On each day, 4 h behavioral observation was made between 9 am and 5 pm. The individual behaviors, *viz*., feeding, drinking, lying down, ruminating, idling, butting (the head pushes forcibly toward the head or shoulders of another goat), pressing (by one goat on another goat’s body), pushing (by anterior part of the goat), frontal clashing (head to head collision), and physical displacement at feed barrier (active and passive: Without physical contact) of the goat were recorded manually using time-sampling method. Further, incidental activities such as defecation and urination were also recorded. Similarly, stereotypic oral activities such as repeatable licking or gnawing of the feeders, walls, wood, and metal were also observed.

### Statistical analysis

Data collected were analyzed by Statistical Analysis System [9] software program version 9.3.

## Results and Discussion

The different ongoing activities after feeding have been documented and presented in the Table-1. The total time spent on feeding by goats in Group I (41.92±1.33 min) was relatively lower as compared to Group II (46.92±1.53 min) and Group III (49.18±0.75 min) and values also differed significantly (p<0.05). However, time spent on feeding did not differ significantly between Group II and Group III. The reason for less time spent in Group I could be due to less possibility of consuming the stem along with leaves. Leaves are stripped off and stems fall on the ground which get soiled in excreta and are not consumed. Hence, less time is spent on feeding. Whereas in Group II, stems are not easily pulled off the linear feeding trough, and some part is consumed. In Group III, availability of chopped fodder leads to indiscriminate consumption of stems along with leaves and the partitioning of feeding trough leads to minimum competition, hence more time spent on feeding.

**Table-1:** Mean cumulative behaviour of kids (min) under different feeding systems at monthly intervals.

Parameters	Group I (min)	Group II (min)	Group III (min)
Feeding	41.92^a^±1.33	46.92^b^±1.53	49.18^b^±0.75
Butting	0.15^a^±0.03	0.11^a^±0.04	0.07^a^±0.02
Feeding with competition	0.77^b^±0.10	0.96^b^±0.11	0.10^a^±0.03
Pushing while feeding	0.48^b^±0.08	0.22^a^±0.04	0.48^b^±0.06
Head to head	0.23^a^±0.03	0.47^a^±0.09	0.45^a^±0.08
Idling	4.67^b^±0.52	1.68^a^±0.21	4.27^b^±0.56
Pushing	0.01^a^±0.01	0.02^a^±0.01	0.00^a^±0.00
Standing near and ruminating	3.33^b^±0.73	1.23^a^±0.49	0.34^a^±0.13
Standing away and ruminating	7.53^b^±0.99	6.99^ab^±1.24	4.19^a^±0.47
Lying away and ruminating	0.00^a^±0.00	1.27^b^±0.62	0.20^ab^±0.06
Defecation	0.08^a^±0.02	0.10^a^±0.03	0.42^b^±0.04
Urinate	0.20^b^±0.03	0.04^a^±0.01	0.27^b^±0.04
Watering	0.01^a^±0.01	0.00^a^±0.00	0.00^a^±0.00

Means having different superscripts row wise differ significantly (p<0.05)

Depending on the composition of the ration and the feeding management, lower-ranking animals might have to eat feed of lower quality when the animals are able to select preferred components [10]. It was observed that Jackfruit had a weak correlation between intake and eating time [4]. The animals consumed almost the same amount per day regardless of time used. The factor dictating intake was not eating time but satiety. In this study, the differences in eating time showed individual differences in eating behavior among animals, influenced by the design of feeder and the form of feed offered.

Butting, head to head, and pushing were the common agonistic behavior found in all the groups, but values did not differ significantly. The tendency to evade conflicts (i.e., low reactivity in response to aggression) or the ability to resist some agonistic contacts (i.e., horn push) could be interpreted as mechanisms that increase the time devoted to feeding by some subordinates, and this tactic is observed in domestic goats [11]. It was reported that there was no effect of the interaction of type of feed barrier and presence of horns or the type of feed barrier itself on the total number of agonistic interactions without physical contact [12] which is in agreement to the present study.

In our study, lack of physical separation seemed to have an unfavorable impact due to the fact that direct physical contact between feeding goats was easily possible. This is confirmed by the highest occurrence of agonistic behavior with physical contact in all groups. It is in agreement with earlier studies [13] who found that more agonistic behavior in a feed barrier type without physical separation (neck rail) as compared to feed barriers with physical separation. Non-transparent head partitions had no effect on feeding behavior and little on agonistic interactions in unrestrained goats [14]. When animals were restrained in the feed barrier, the number of agonistic interactions was lower and feeding scans higher, especially in low-ranking horned goats, with head partitions [14]. Offering fixed feeding places induces a definite distance between the necks of the animals and can reduce agonistic interactions [11], which was seen in Group III. Feed barriers used for dairy goats often provide feeding spaces of about 35-45 cm per goat which are clearly smaller than the individual distance required by most goat dyads during feeding [15].

Pushing while feeding was relatively less in Group II (0.22±0.04 min) which differed significantly with other two groups, but values between Group I (0.48±0.08 min) and Group III (0.48±0.06 min) did not differ significantly. The idling time was significantly (p<0.05) less in Group II (1.68±0.21 min) in comparison to Group I (4.67±0.52 min) and Group III (4.27±0.56 min). Values of Group I and Group III did not differ significantly with each other. Time spent in rumination near the feeding trough as well as away from feeding trough was significantly higher in Group I as compared to Group II and Group III. Since less time is spent on feeding, more time is spent on ruminating. There was no significant difference between Group II and Group III. Other minor activities, *viz*., defecation and urination were negligible. No stereotypic activities were observed. The goats of Group I spent less time in feeding as well in agonistic behavior. Further, these goats also spent more time in idling and rumination. The possible reason could be that goats tried to consume only foliage due to selective process. While consuming the leaf part, goats tried to strip parallel which further led to fall of stem part out of the feeding trough on the ground ultimately leading to wastage of stem portion. Goats find it difficult to eat directly off the ground. As goats are selective feeders by natural habit, they do not eat once the feed is dropped on the ground and stamped [1].

## Conclusion

It was concluded that provision of un-chopped fodder in circular feeder did not give any advantage over linear feeding though it simulated the natural feeding behavior of goats and helped to exploit the natural feeding instinct of goat. Further, feeding chopped fodder in linear feeding trough not only lead to increased consumption but also more time is spent on feeding than on agonistic behavior as compared to the other two groups.

## Authors’ Contributions

BHMP and AK jointly planned and designed the study. AK performed the study and wrote the manuscript. VM, MS, and ASG assisted in the execution of the study. MRV helped in statistical analysis. All authors read and approved the final manuscript.
